# Delayed esophageal perforation following lightning strike: a case report and review of the literature

**DOI:** 10.1186/1752-1947-6-244

**Published:** 2012-08-20

**Authors:** Patricia Figgis, George Alvarez

**Affiliations:** 1 The Prince of Wales Hospital, Randwick, NSW 2031, Sydney, Australia; 2 Rockyview General Hospital, Alberta Health Services, 7007-14th Street SW, Calgary, AB, T2P 1P9, Canada

## Abstract

**Introduction:**

Lightning is the second most common storm-related cause of death. The mortality following lightning strike is 10% to 30% and a large proportion of these people suffer cardiopulmonary arrest at the time of the strike. Much less commonly, solid organ injuries occur from either primary or secondary blunt force trauma.

**Case presentation:**

To the best of our knowledge, this is the first case report in the literature of an isolated esophageal rupture caused by lightning strike blunt force trauma.

**Conclusions:**

Solid organ injuries are often underappreciated in lightning strikes. Blast injury to patients by lightning strikes should prompt clinicians to search for occult organ injury.

## Introduction

Lightning is the second most common storm-related cause of death, exceeded only by flash floods. The mortality following lightning strike is 10% to 30% with a bias towards the reporting of fatalities. A large proportion of the fatalities experience either ventricular fibrillation or an asystole cardiopulmonary arrest at the time of the strike. The injuries seen in survivors include neurological phenomenon, cardiac dysrhythmia, vasospasm, burns, and tympanic and ocular damage [1]. Much less commonly, solid organ injuries occur from either primary or secondary blunt force trauma. To the best of our knowledge, this is the first case in the literature of an isolated esophageal rupture caused by lightning strike blunt force trauma.

## Case presentation

Our patient was a 79-year-old Caucasian man who was hit by the ground current of a lightning strike while holding onto the metal railing of his veranda. He was thrown back two meters and suffered a fractured right humerus but never lost consciousness. He was admitted to a regional hospital for observation with casting of his fractured arm and subsequently discharged. Four days later, our patient complained of acute upper abdominal pain followed by an episode of hematemesis. At re-presentation, he had hemodynamic instability and was hypoxic, requiring intubation. Computed tomography (CT) scans of his chest and abdomen demonstrated bilateral hydrothorax with esophageal rupture and, therefore, bilateral chest drains were inserted. He was transferred to our institution for a surgical opinion and management of his septic shock with multiorgan failure.

**Figure 1:**
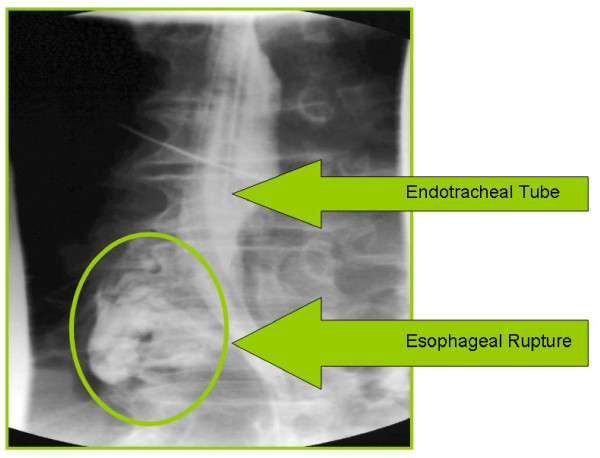
Intubated patient showing esophageal rupture during gastrografin swallow.

**Figure 2:**
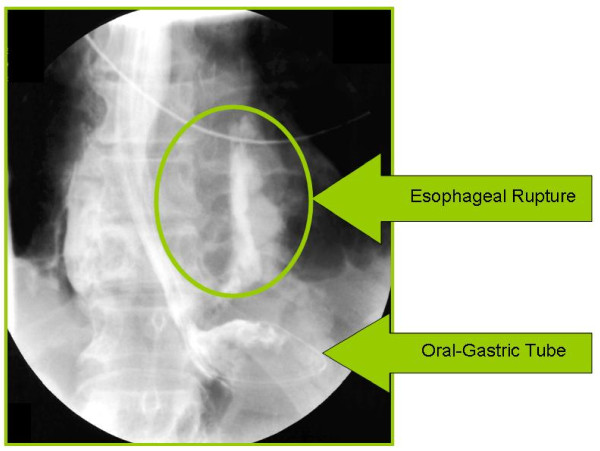
Posterior view of the gastrografin series.

A transesophageal echocardiogram showed normal left ventricular size and function but markedly dilated right-sided chambers with severe tricuspid regurgitation and no aortic dissection. Because of his hemodynamic instability and uncertainty of survival, it was elected to treat our patient non-surgically. He was managed with inotropic support, broad-spectrum antibiotics, further pleural space drainage, total parental nutrition and renal replacement therapy. On the 12^th^ day of admission, a CT-gastrografin swallow demonstrated a persistent distal esophageal rupture into the pleural space (Figure1 and 2). A total esophagectomy with decortication of his right pleural space and insertion of a jejunostomy feeding tube was performed on the 21^st^ day. Unfortunately, our patient’s condition worsened and he died eight days after surgery.

## Discussion

Lightning cannot be classified as either a direct or alternating current, its physics are extremely complex and differ from generated electricity. It can release greater than 1 million volts of energy and can generate currents of greater than 200,000 A. The damage caused is determined by the strength of the current (which is directly proportional to the source voltage and inversely proportional to the resistance of the conducting medium) and the duration of the application of the energy. The pathway that a current follows determines the type of injury, the tissues involved and the extent of conversion to heat energy.

In contrast with high voltage electrocution injuries, severe burns and solid organ injuries are unusual because of the very short duration (2 milliseconds) of current flowing through the body and a phenomenon known as flashover, where the majority of the energy flows externally over the body. Injuries caused by lightning strike are thought to occur by five different mechanisms; direct strike, contact injury, side splash, ground current and blunt trauma [2,3].

A direct strike occurs when the lightning hits the person directly, usually on or near the head. However, a proportion of the energy may enter orifices such as the ears and flow internally, mostly through the neurological and vascular systems. Contact injury occurs when the victim touches an object within the lightning pathway. Side splash injury occurs when lightning jumps from the primary strike site on to the victim. Ground current injury occurs when a lightning strike hits the ground, spreads radially, and enters the victim’s feet. Blunt trauma can either be primary or secondary (examples of the latter include falls, airborne debris or infrastructure collapse). Primary blunt force trauma can be caused by two mechanisms. Firstly, the current flow may cause a muscular contraction, throwing the patient. Secondly, a blast wave may be produced with massive and sudden fluctuations in temperature. Air surrounding and within the victim is very rapidly super-heated to temperatures as high as 27,760°C (50,000°F) and almost instantaneously expands and contracts, creating a shockwave that can throw the victim and/or cause blast wave injuries. Examples of blast wave injuries include myocardial and pulmonary contusions, ruptured lung, shearing of large vessels, ruptured tympanic membrane, ocular damage and intestinal rupture. These types of injuries are well described and reproducible in bomb blasts and experimental models [4]. Our patient’s severe right heart abnormalities are likely attributable to the blunt force contusion injury.

While tympanic rupture and ocular damage are well documented in people experiencing a lightning strike , there is limited literature on solid organ blast wave injuries. There are two case reports describing patients with a lightning-related severe blast injury to the lungs, and a third describing an isolated pneumomediastinum presumably secondary to alveolar rupture [5-7]. Postmortem studies of people killed by a lightning strike have shown a significant proportion of blast injuries, and the same authors demonstrated blast injuries to the lungs and other solid organs using artificial lightning in animal models [8].

The reader may wonder if our patient had an occult injury that is known to be associated with esophageal injury, for example, cervical spine fracture or Boerhaave’s syndrome [9,10]. We could not identify any other injury through our patient’s medical history or diagnostic imaging. Furthermore, esophageal rupture tends to present acutely with pain, like in the instance of our patient four days after the lightning strike. Esophageal rupture is a rare, difficult diagnosis to make and an even more challenging syndrome to treat. Highly specialized institutions spend decades collecting data to achieve modest case series [11,12]. Various medical, surgical and minimally invasive procedures exist that are beyond the scope and purpose of this case report [13,14].

## Conclusions

Based on this case and our review of the available literature, it would seem prudent to have a high index of suspicion of blast injury in patients who have experienced a lightning strike, and the presence of one such injury should prompt careful examination for others. Blast injury is an important cause of morbidity and mortality in lightning strikes.

## Consent

Written informed consent was obtained from the patient’s family for publication of this case report and any accompanying images. A copy of the written consent is available for review by the Editor-in-Chief of this journal.

## Competing interests

The authors declare that they have no competing interests.

## Authors’ contributions

GA and PF contributed equally to the final manuscript. Both authors read and approved the final manuscript.

## References

[B1] O'Keefe Gatewood M Zane RD Lightning injuries Emerg Med Clin North Am 2004 22 369 403 10.1016/j.emc.2004.02.002 15163573

[B2] Strasser EJ Davis RM Menchey MJ Lightning injuries J Trauma 1977 17 315 319 10.1097/00005373-197704000-00010 857050

[B3] Cooper MA A fifth mechanism of lightning injury Acad Emerg Med 2002 9 172 174 10.1111/j.1553-2712.2002.tb00237.x 11825846

[B4] Mellor SG The pathogenesis of blast injury and its management Br J Hosp Med 1988 39 536 539 3395754

[B5] Moulson AM Blast injury of the lungs due to lightning Br Med J (Clin Res Ed) 1984 289 1270 1271 10.1136/bmj.289.6454.1270 PMC1443487 6437514

[B6] Soltermann B Frutiger A Kuhn M Lightning injury with lung bleeding in a tracheotomized patient Chest 1991 99 240 242 10.1378/chest.99.1.240 1984964

[B7] Halldorsson A Couch MH Pneumomediastinum caused by a lightning strike J Trauma 2004 57 196 197 10.1097/01.TA.0000119167.63219.11 15284576

[B8] Ohashi M Hosoda Y Fujishiro Y Tuyuki A Kikuchi K Obara H Kitagawa N Ishikawa T Lightning injury as a blast injury of skull, brain, and visceral lesions: clinical and experimental evidences Keio J Med 2001 50 257 262 10.2302/kjm.50.257 11806503

[B9] Nérot C Jeanneret B Lardenois T Lépousé C Esophageal perforation after fracture of the cervical spine: case report and review of the literature J Spinal Disord Tech 2002 15 6 513 518 10.1097/00024720-200212000-00014 12468980

[B10] Griffiths EA Yap N Poulter J Hendrickse MT Khurshid M Thirty-four cases of esophageal perforation: the experience of a district general hospital in the UK Dis Esophagus 2009 22 7 616 625 10.1111/j.1442-2050.2009.00959.x 19302220

[B11] Vallböhmer D Hölscher AH Hölscher M Bludau M Gutschow C Stippel D Bollschweiler E Schröder W Options in the management of esophageal perforation: analysis over a 12-year period Dis Esophagus 2010 23 3 185 190 10.1111/j.1442-2050.2009.01017.x 19863642

[B12] Eroglu A Turkyilmaz A Aydin Y Yekeler E Karaoglanoglu N Current management of esophageal perforation: 20 years experience Dis Esophagus 2009 22 4 374 380 10.1111/j.1442-2050.2008.00918.x 19207557

[B13] Chirica M Champault A Dray X Sulpice L Munoz-Bongrand N Sarfati E Cattan P Esophageal perforations J Visc Surg 2010 147 3 117 128 10.1016/j.jviscsurg.2010.08.003 20833121

[B14] Leers JM Vivaldi C Schäfer H Bludau M Brabender J Lurje G Herbold T Hölscher AH Metzger R Endoscopic therapy for esophageal perforation or anastomotic leak with a self-expandable metallic stent Surg Endosc 2009 23 10 2258 2262 10.1007/s00464-008-0302-5 19184216

